# Identifying research priorities for conducting dementia‐related research with ethno‐racial communities: Results of a DELPHI study

**DOI:** 10.1002/alz70858_101660

**Published:** 2025-12-25

**Authors:** Carrie A McAiney, Dana Zummach, Kodikara Perera, Christine Pellegrino, the Dementia & Diversity Steering Committee

**Affiliations:** ^1^ Schlegel‐UW Research Institute for Aging, Waterloo, ON, Canada; ^2^ University of Waterloo, Waterloo, ON, Canada

## Abstract

**Background:**

The population of people living with dementia, globally and within many countries, is becoming increasingly ethno‐racially diverse. Yet, research in dementia does not adequately reflect this diversity. Guidance on research priorities can help researchers make decisions about the areas of study on which they want to focus. Engaging people living with dementia and family/friend care partners – particularly those who are part of ethno‐racial communities – is critical to ensuring that the research priorities identified align with the needs and preferences of individuals impacted by dementia who are from ethno‐racial communities. Thus, a DELPHI study with a diverse group of participants was undertaken to identify research priorities in dementia and ethno‐racial diversity.

**Method:**

Multiple strategies were used to identify the items to include in the DELPHI surveys and the members of the DELPHI panel. Survey items: We gathered suggestions at a symposium on dementia and ethno‐racial diversity. Attendees included people living with dementia, care partners, providers who work with people living with dementia and/or people from diverse communities, and researchers. These suggestions were supplemented with gaps identified in the literature. The generated items (*N* = 45) were grouped into 9 categories. DELPHI panel: participants in previous projects related to diversity were invited to participate. Snowball sampling was used to identify additional members. In total, 74 individuals were invited to participate. Three rounds of surveys were administered with participants rating the importance of each item (1=not important, 5=extremely important). In the final round, participants identified their top 5 most important research questions and 2 most important categories of research questions.

**Result:**

Average ratings in Round 1 ranged between 3.71 and 4.78. No items were dropped following Round 1. Average ratings in Round 2 were similar, ranging from 3.47 to 4.84. Most of the top‐rated items related to health care and health service use followed by well‐being.

**Conclusion:**

The high ratings of importance across items suggests that the significant gaps in this area makes differentiating among priorities challenging. Nevertheless, the identified research priorities can inform researchers interested in research on dementia and ethno‐racial diversity.

